# Differential target multiplexed SCS (DTM SCS): a rational hypothesis

**DOI:** 10.1093/pm/pnag041

**Published:** 2026-06-10

**Authors:** Leonardo Kapural, Ricardo Vallejo, David L Cedeño, Krishnan Chakravarthy, Jan Willem Kallewaard, Alaa Abd-Elsayed, Michael A Fishman, Melissa Murphy, Julia E Gamache, Lisa M Johanek

**Affiliations:** Carolinas Pain Institute, Winston-Salem, NC, United States; Lumbrera LLC, Department of Research and Development, Bloomington, IL, United States; Lumbrera LLC, Department of Research and Development, Bloomington, IL, United States; VA San Diego Healthcare, San Diego, CA, United States; Innovative Pain Treatment Solutions and Surgery Centers, San Diego, CA, United States; Solaris Research Institute, San Diego, CA, United States; Rijnstate Hospital, Department of Anesthesiology and Pain Management, Arnhem, Netherlands; Amsterdam University Medical Center, Amsterdam, Netherlands; University of Wisconsin, Department of Anesthesia, Madison, WI, United States; Michael Fishman MD, PLLC, Lebanon, PA, United States; Brixton Biosciences, Cambridge, MA, United States; North Texas Orthopedics & Spine Center, Grapevine, TX, United States; Medtronic Neuromodulation, Minneapolis, MN, United States; Medtronic Neuromodulation, Minneapolis, MN, United States

**Keywords:** spinal cord stimulation, differential target multiplexed, chronic pain, back pain, FBSS (failed back surgery syndrome), neuropathic pain, waveforms, upper limb pain

## Abstract

**Background:**

Early in the development of technology for spinal cord stimulation (SCS) therapy, the challenge of treating chronic low back pain was met with specific electrode configurations and programming parameters designed to target and maintain paresthesia in that region. New modalities were subsequently developed to achieve long-term relief of chronic low back and leg pain and minimize unwanted stimulation. Only recently has it become clear that SCS mechanisms for modulating pain involve not only the activation of Aβ-fibers but also other mechanisms, such as the modulation of biological processes at the glial cells and neuroglial interaction.

**Content overview:**

Differential Target Multiplexed^TM^ (DTM^TM^) programming, a combination of multiplexed pulsed signals delivered synchronously at different rates and amplitudes, was shown in animal models to revert neurons and glia in the stimulated spinal cord from an injured state back to a noninjured state in transcriptomics and proteomics studies. This article highlights the rationale for and development of DTM SCS and provides an overview of its clinical application. We review key outcomes of pivotal DTM SCS studies within the context of other randomized clinical trials (RCTs) examining SCS for the treatment of chronic low back pain.

**Conclusions:**

The development of DTM SCS is based on preclinical research, parameter refinement in a feasibility study, and confirmatory RCTs showing promise in enhancing outcomes of patients with chronic low back pain.

## Historical perspective

Spinal cord stimulation (SCS) therapy was developed as a treatment for various types of chronic neuropathic pain. Early evidence supported the use of SCS for the treatment of chronic low back and leg pain related to prior spine surgery—conditions referred to as post-laminectomy pain, failed back surgery syndrome (FBSS), or persistent spinal pain syndrome Type 2 (PSPS-T2).^1^ More recent evidence supports the use of SCS in patients with chronic refractory back pain with no surgical indication, including those diagnosed with degenerative disc disease and radicular pain, and do not have a history of prior spine surgery or persistent spinal pain syndrome Type 1 (PSPS-T1). Pain patterns in both PSPS-T1 and -T2 are heterogeneous: some patients experience predominantly leg pain, others predominantly back pain, and some a combination of both. Foundational randomized controlled trials (RCTs) comparing SCS to conventional medical management (CMM)^2^ or reoperation^3^ in patients with PSPS-T2 focused on the radicular component of pain when demonstrating the effectiveness of SCS therapy.

As SCS was increasingly used for PSPS-T2, clinicians observed that targeting and maintaining paresthesia coverage of the low back was challenging. Various electrode configurations were explored, including bipole configurations,^4^ multi-column leads and side-by-side leads,^5^ guarded longitudinal cathode configurations,^6^ and transverse tripole configurations.^7^ Later strategies focused on widening pulse widths up to 1000 μs to increase paresthesia coverage^8^^,^^9^ or adjusting the frequency of stimulation for broader coverage.^10^ Interestingly, a study focused on varying frequency between 40 and 1200 Hz, while holding the pulse width at 300 μs, showed that the threshold for perception, therapy, and discomfort levels were inversely proportional to the frequency, indicating that higher frequencies lowered the threshold for sensation.^11^

Surgical leads were also considered to improve and stabilize low back targeting. Several feasibility studies suggested surgical leads would accomplish paresthesia coverage of the low back^12^^,^^13^; however, an RCT demonstrated only moderate relief of low back pain using a specific surgical lead at the expense of a laminotomy and possible additional surgical and post-surgical complications.^14^ The increasing complexity of device programming and the need for improved low back pain relief led to the development of computer-assisted, algorithm-based programming. One study evaluated a 3-dimensional programming algorithm that precisely calculated the required current for independent, current-controlled contacts using anatomically guided neural targeting.^15^ This study included a population of patients (*n* = 89) with chronic, axial back pain and no radicular component and reported a responder rate (≥50% reduction in pain) of 58% at the primary endpoint.^15^

**Figure 1:**
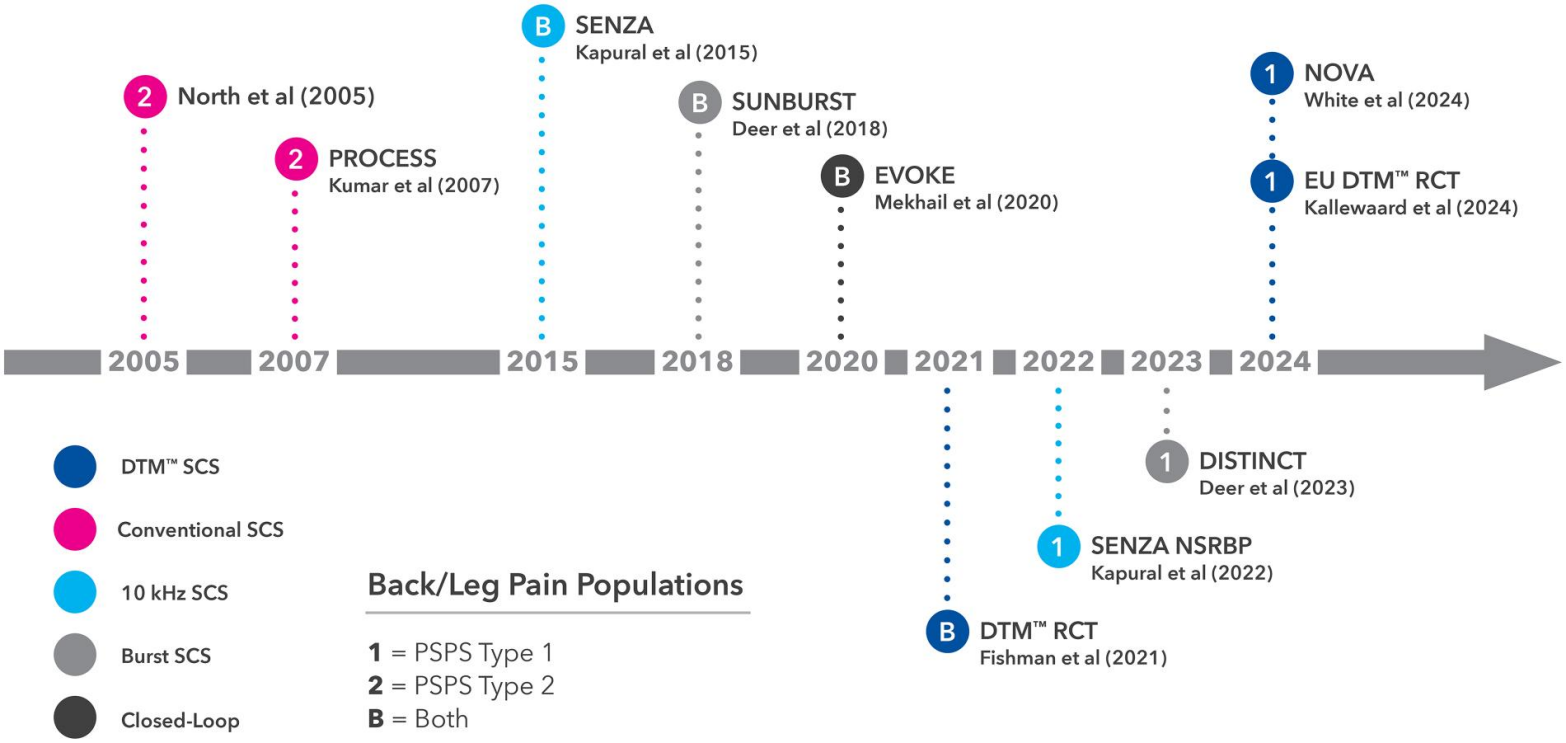
Timeline of RCTs investigating SCS modalities related to patients with PSPS-T1 (labeled as “1”) and PSPS-T2 (labeled as “2”). “B” indicates the study enrolled both PSPS-T1 and PSPS-T2 indications. Foundational RCTs used conventional SCS programming, typically with frequencies below 100 Hz, to investigate pain relief in patients with PSPS-T2.^2^^,^^3^ Robust back pain relief was demonstrated using 10 kHz and 30 μs in the SENZA and SENZA non-surgical refractory back pain (NSRBP) studies for PSPS Type 2 (86.6% of patients were PSPS-T2) and PSPS-T1, respectively.^26^^,^^27^ The SUNBURST study investigated the effect of a specific burst pattern, BurstDR, on patients with back and leg pain.^28^ BurstDR consists of 5 pulses at 500 Hz with a 1000 μs pulse width; each burst repeats at a frequency of about 40 Hz with passive recharge. Closed-loop stimulation of low-frequency SCS also improved back pain in PSPS-T1 and PSPS-T2 patients in the EVOKE study.^29^ Patients with PSPS-T1 obtained pain relief with BurstDR in the DISTINCT study.^30^ DTM SCS is further described in this article, but generally consists of multiplexing a low-frequency base signal around 50 Hz and high-frequency prime signals between 200 and 1200 Hz. Patients with back and leg pain showed robust pain relief in the DTM SCS RCT; patients with PSPS Type 1 were further studied in the NOVA^31^ and European DTM SCS RCT.^32^

Long-term, effective low back pain relief was ultimately achieved with the emergence of new waveforms, or modalities, of stimulation (Figure 1).^16^ Unlike conventional SCS therapy, which relied on strong paresthesia coverage, these new modalities tended to be programmed below the perception level or without paresthesia. Since conventional SCS could cause uncomfortable stimulation, these subperception options were pursued as an appealing alternative approach.^17^ However, the mechanism of action remains controversial, as the lack of paresthesia challenges the gate control theory of Aβ-fiber activation. Although the higher energy input with higher frequencies or wider pulse widths^18^ used by these modalities demonstrated robust relief of low back pain and responder rates,^19^ the precise clinical mechanisms are unclear. Hypotheses for pain relief have included direct inhibition of dorsal horn neurons^20^ and burst-like activation of the medial pain-signaling pathway.^21^ Many review articles have discussed the research supporting various waveforms.^22–25^

In recent years, clinical studies have highlighted the effectiveness of diverse SCS modalities in achieving sustained relief for patients with low back pain. At least 3 studies investigating patients with various low back and leg pain indications have reported responder rates (≥50% reduction in pain) specifically for low back pain at 12 months: the SENZA RCT (77% responder rate),^19^ the EVOKE RCT (80% responder rate),^29^ and the DTM SCS RCT (84% responder rate).^33^ Studies investigating patient populations categorized as PSPS-T1 have reported responder rates up to 88% at 24 months.^32^^,^^34^ Together, these advances have shaped a dynamic and evolving landscape for SCS therapy, with ongoing innovations aimed at improving outcomes for a broader range of chronic pain conditions. Among these developments, the differential target multiplexed (DTM) SCS algorithm has emerged as a promising approach within the field. DTM SCS therapy is based on 2 core principles: spatially distinct anatomical targeting (“differential targeting”) and the simultaneous delivery of multiple electrical signals (“multiplexing”). By using specific electrode configurations, DTM SCS can stimulate different spinal cord regions concurrently, forming the foundation of its programming approach.

This article reviews the development pathway of the DTM SCS algorithm, from preclinical testing to optimization in a clinical feasibility study. It provides an overview of the key findings in the DTM SCS RCT and describes the results within the context of the current clinical evidence RCT landscape for SCS therapy. Finally, the continued use and investigation of DTM SCS in 2 RCTs for indicated PSPS-T1 patients with low back pain, degenerative disc disease, and radicular pain, who are not candidates for spine surgery, is discussed. Clinical application of DTM SCS for chronic upper limb pain (for indicated patients, eg, radiculopathy or complex regional pain syndrome) is also included.

## DTM SCS: preclinical foundation

Historical research has established that SCS acts through multiple mechanisms, initially focusing on the activation of Aβ-fibers and the gate control theory of pain.^23^ Over time, research expanded to include inhibition of wide dynamic range neurons, brainstem and descending inhibition, cortical modulation, interaction with the sympathetic nervous system, and the involvement of neurotransmitters and neuromodulators such as GABA, glycine, endogenous opioids, and cannabinoids.^35^^,^^36^ Much of this research was predicated on the gate control theory of pain,^37^ which presumed that activation of Aβ-fibers was crucial for SCS effectiveness. However, subsequent clinical studies demonstrated that effective pain relief could be achieved with little or no paresthesia, challenging the centrality of Aβ-fiber activation. Consequently, research shifted to investigating higher frequencies of stimulation at amplitudes below the threshold for Aβ-fiber activation.^38–40^ The putative mechanisms behind subthreshold stimulation are still unclear but may involve modulation of local axon terminals or direct dorsal horn modulation.^22–25^^,^^41^

Glial cells, though historically overlooked in SCS research, are now recognized for their role in chronic pain modulation in both preclinical^35^^,^^36^ and clinical studies.^42^ Electrical stimulation of the spinal cord has been shown to modulate glial cell activity in preclinical studies.^43–45^ However, whether specific stimulation parameters can be optimized to target glial cells—while also affecting neuronal mechanisms—to enhance pain relief remains unclear. This represents a key gap in understanding the potential to fine-tune SCS for optimal therapeutic outcomes.

Studies have evaluated the effects of multiplexed stimulation signals—specifically, components at 50 Hz (150 µs) and 1200 Hz (50 µs) delivered via a 4-contact lead—on neuronal and glial modulation in a rat spared nerve injury (SNI) model of neuropathic pain.^46^ This preclinical application, termed differential target multiplexed programming (DTMP), significantly reversed mechanical hypersensitivity to a greater extent than low rate (LR; 50 Hz) or high rate (HR; 1200 Hz) stimulation alone, and significantly reversed heat and cold hypersensitivity relative to baseline measurements.^46^ These findings suggest that DTMP may engage distinct or additional mechanisms of action to enhance pain relief compared to conventional stimulation paradigms. Other studies demonstrated that the way recharge balance is achieved (active versus passive, symmetric versus asymmetric),^47^ and different amplitudes of the same signals generate distinct patterns of transcript expression.^48^

Initial mechanistic insights into DTMP were obtained through RNA sequencing of stimulated spinal cord tissue, followed by analyses of measured gene expression patterns. These transcriptomic studies provide a comprehensive view of the genes affected by a nerve injury and the influence of neuromodulation on gene expression. By categorizing genes according to biological processes, function, or cell type, the potential mechanisms being engaged by DTMP have been further described. Overall, DTMP modulated a greater number of pain-related genes, returning the expression levels closer to those observed in the naïve, noninjured animals compared to LR or HR SCS.^46^ Cell type-specific analysis revealed that DTMP produced gene expression profiles in neurons, microglia, astrocytes, and oligodendrocytes that closely matched those of healthy, naïve state animals.^49^ In contrast, HR SCS only correlated strongly with naïve state gene expression in microglia, while LR SCS showed no strong correlation in any cell type. These findings support the hypothesis that DTMP promotes normalization of gene expression across multiple cell types, potentially involving modulation of microglial activation and associated pro- and anti-inflammatory processes.^50^

Ongoing research has demonstrated the impact of DTMP on protein and phosphoprotein expression in pathways related to inflammation,^51^ ion transport,^52^ signaling,^53^ and extracellular matrix processes.^54^ DTMP is also being investigated in other types of injury models, including streptozotocin-induced painful diabetic peripheral neuropathy in rats, where DTMP maintained its ability to shift gene expression levels toward an anti-inflammatory state.^55^ Finally, appreciating the need to evaluate these mechanisms in the context of delivering subthreshold stimulation, Cedeno et al^48^ reported similar effects of DTMP at 40% and 70% motor threshold in reversing pain behaviors and influencing gene expression in neurons and glial cells.

Preclinical investigations have yielded important insights into cell type-specific mechanisms of chronic pain (Table 1), forming the foundation for translating DTMP into the clinical application of DTM SCS. Currently, DTMP is the only waveform shown to optimally modulate glia and neurons at both a transcriptomic and proteomic level in preclinical research. Despite these advances, no clear dose-response relationship has been established for DTMP, and the mechanisms by which stimulation relieves pain remain unclear and are likely multifactorial. Pain is a complex emotional and sensory experience. While preclinical models—including transcriptomic profiling and nociceptive assays—offer valuable mechanistic clues, they cannot fully capture the subjective and multidimensional nature of pain relief. A more comprehensive understanding requires integration of preclinical and clinical mechanism research with clinical outcomes.

**Table 1:** DTMP preclinical and mechanisms research.

Citation	Brief overview	Study design
Vallejo et al (2020)^46^	DTMP showed greater reversal of pain behaviors in a rodent SNI pain model and modulated more genes back toward the noninjury state compared to LR SCS or HR SCS.	Model: SNI rodent model of neuropathic pain Stimulation: LR = 50 Hz, 150 µs; HR = 1200 Hz, 50 µs; DTMP = 50 Hz, 150 µs and 1200 Hz, 50 µs distributed over 4 contacts; ∼70% MT; 48 hours of stimulation Assay: Paw withdrawal threshold testing; SC tissue collection for RNA and WGCNA analysis Result: DTMP SCS showed greater reversal of pain behaviors and modulated more genes back toward the noninjury state compared to LR or HR.
Cedeno et al (2020)^49^	Gene expression after DTMP SCS in a rodent SNI pain model strongly correlated with that of healthy animals across all cell types (neurons, microglia, astrocytes, and oligodendrocytes). In contrast, gene expression after HR SCS only showed a strong correlation in the microglia transcriptome and LR SCS showed no strong correlation with healthy-state gene expression.	Model: SNI rodent model of neuropathic pain Stimulation: LR = 50 Hz, 150 µs; HR = 1200 Hz, 50 µs; DTMP = 50 Hz, 150 µs and 1200 Hz, 50 µs distributed over 4 contacts; ∼70% MT; 48 hours of stimulation Assay: SC tissue collection for RNA analysis of transcriptomes specific to neurons, microglia, astrocytes, and oligodendrocytes. Result: Gene expression after DTMP SCS strongly correlated with that of healthy animals across all cell types; in contrast, gene expression after HR only showed a strong correlation in the microglia transcriptome and LR showed no strong correlation with healthy-state gene expression.
Smith et al (2021)^50^	Across all microglia transcriptomes (resting, post-injury, neuroprotective/repopulation), gene expression after DTMP in a rodent SNI pain model was most strongly correlated to the healthy animals compared to HR SCS or LR SCS.	Model: SNI rodent model of neuropathic pain Stimulation: LR = 50 Hz, 150 µs; HR = 1200 Hz, 50 µs; DTMP = 50 Hz, 150 µs and 1200 Hz, 50 µs distributed over 4 contacts; ∼70% MT; 48 hours of stimulation Assay: SC tissue collection for RNA analysis of transcriptomes specific to microglia activation and pro- and anti-inflammatory expression patterns. Result: Across all microglia transcriptomes (resting, post-injury, neuroprotective/repopulation), gene expression after DTMP was most strongly correlated to the healthy animals compared to HR or LR.
Cedeno et al (2022)^51^	SNI pain model in rodents increased expression levels of SC proteins in pathways related to inflammation; these increases were reversed by DTMP. LR SCS also modulated inflammation-related proteins, but to a lesser extent.	Model: SNI rodent model of neuropathic pain Stimulation: LR = 50 Hz, 150 µs; DTMP = 50 Hz, 150 µs and 1200 Hz, 50 µs distributed over 4 contacts; ∼70% MT; 48 hours of stimulation Assay: The impact of SCS on SC proteins associated with MAP kinases and NFƙB-signaling relevant to neuroinflammation was investigated using liquid chromatography/tandem mass spectrometry Result: The SNI pain model increased expression levels of proteins in the complement pathway, NFƙB-signaling related proteins, and phosphorylated protein kinases; these increases were reversed by DTMP. LR SCS also modulated inflammation-related proteins, but to a lesser extent compared to DTMP.
Tilley et al (2022)^52^	The study identified over 40 proteins significantly involved in the regulation of ion transport in animals with an SNI pain model. Specific proteins were modulated by the nerve injury as well as by DTMP.	Model: SNI rodent model of neuropathic pain Stimulation: LR = 50 Hz, 150 µs; DTMP = 50 Hz, 150 µs and 1200 Hz, 50 µs distributed over 4 contacts; ∼70% MT; 48 hours of stimulation Assay: Expression levels of SC proteins specifically involved in ion transport regulation were identified using bioinformatic tools and compared between rodents using liquid chromatography/tandem mass spectrometry Result: The study identified over 40 proteins significantly involved in the regulation of Cl−, K+, Na+, or Ca2+ ions. Specific proteins were modulated by nerve injury as well as by DTMP.
Tilley et al (2022)^53^	Phosphoproteins related to mTOR signaling affected by the SNI pain model were reversed by 66% following DTMP and by 58% following LR SCS.	Model: SNI rodent model of neuropathic pain Stimulation: LR = 50 Hz, 150 µs; DTMP = 50 Hz, 150 µs and 1200 Hz, 50 µs distributed over 4 contacts; ∼70% MT; 48 hours of stimulation Assay: Expression levels of SC proteins related to the mTOR signaling pathway were identified using bioinformatic tools and compared between rodents using liquid chromatography/tandem mass spectrometry. Result: A total of 1451 and 705 proteins were affected by DTMP and LR-SCS, respectively (*P* < .05). Phosphoproteins related to mTOR (*n* = 119) affected by the injury model were reversed by 66% following DTMP and by 58% following LR-SCS.
Cedeno et al (2023)^48^	DTMP SCS at a lower amplitude (40% motor threshold, MT) compared to 70% MT resulted in similar reversal of pain behaviors and RNA expression in neurons and glial cells in rodents with an SNI pain model.	Model: SNI rodent model of neuropathic pain Stimulation: DTMP = 50 Hz, 150 µs and 900 Hz (3, 300 Hz programs), 50 µs distributed over 4 contacts; either 40% or 70% MT; 48 hours of stimulation Assay: Paw withdrawal threshold testing; SC tissue collection for RNA expression analysis. Result: DTMP at 40% MT compared to 70% MT resulted in similar reversal of pain behaviors and RNA expression in neurons and glial cells.
Tilley et al (2023)^54^	DTMP reversed the expression levels of extracellular matrix proteins back toward the uninjured state. In contrast, LR SCS reversed fewer extracellular matrix proteins back toward normal levels.	Model: SNI rodent model of neuropathic pain Stimulation: LR = 50 Hz, 150 µs; DTMP = 50 Hz, 150 µs and 1200 Hz, 50 µs distributed over 4 contacts; ∼70% MT; 48 hours of stimulation Assay: Expression levels of extracellular matrix SC proteins were identified using bioinformatic tools and compared between rodents using liquid chromatography/tandem mass spectrometry. Result: DTMP reversed the expression levels of 83% extracellular matrix proteins back toward the uninjured state. In contrast, LR reversed 67% of extracellular matrix proteins.
De Geus et al (2023)^55^	All stimulation types decreased mechanical hypersensitivity in streptozotocin-induced painful diabetic peripheral neuropathy in rats. LR SCS increased specific pro-inflammatory cytokines, while HF SCS and DTMP tended to shift expression levels towards an anti-inflammatory state.	Model: Streptozotocin-induced painful diabetic peripheral neuropathy in rats Stimulation: LR = 50 Hz, 150 µs; HR = 1200 Hz, 50 µs; DTMP = 50 Hz, 150 µs and 1200 Hz, 50 µs distributed over 4 contacts; 50% MT; 48 hours of stimulation Assay: Rodent behavior was assessed through paw withdrawal threshold testing and a mechanical conflict avoidance test. RNA expression of pro- and anti-inflammatory cytokines and markers of glial cell activation were studied in spinal cord tissue. Result: All stimulation types decreased mechanical hypersensitivity after 48 hours of stimulation. 50 Hz SCS increased specific pro-inflammatory cytokines, while HF and DTMP tended to shift expression levels towards an anti-inflammatory state.

Abbreviations: Ca2+, calcium ion; Cl−, chloride; DTMP, differential target multiplexed programming; HR, high rate; Hz, Hertz; K+, potassium ion; LR, low rate; MAPK, mitogen-activated protein kinase; MT, motor threshold; µs, microsecond; Na+, sodium ion; NFƙB, nuclear factor kappa-light-chain-enhancer of activated B cells; RNA, ribonucleic acid; SC, spinal cord; SCS, spinal cord stimulation; SNI, spared nerve injury; WGCNA, weighted gene co-expression network analysis.

## DTM SCS clinical feasibility

To translate preclinical findings into clinical practice, a feasibility study was conducted to refine and optimize the preclinical DTMP approach for patient use. While preclinical models offer valuable insights into neuropathic pain and therapy mechanisms, they present limitations; anode and cathode configurations, spinal targets, and optimal amplitudes cannot be determined in small animal models. Moreover, in this feasibility study, patients trialed multiple DTM SCS program combinations to systematically identify optimal initial parameters, enabling the development of a standardized approach for future clinical studies.

The study enrolled 25 patients with chronic intractable low back pain, with leg pain equal to or less than back pain. Eligible participants had a pain score of ≥5 on the 11-point numeric rating scale (NRS) and were approved for SCS screening. The trial utilized a 1-way crossover design: all subjects first underwent conventional SCS programming (20-200 Hz) for 3-5 days, followed by a washout period and fluoroscopic confirmation of lead placement. Subsequently, each patient was offered up to 8 distinct DTM SCS programs, featuring charge-balanced pulses with stimulation frequencies between 50 and 1200 Hz and pulse widths ranging from 50 to 400 µs. Electrodes were positioned between T8 and T11, and patients adjusted amplitudes for optimal pain relief. Participants evaluated 1-2 programs per day and ultimately selected the program that provided the greatest relief from low back pain.^56^

The primary outcome was the change in low back pain NRS relative to baseline after conventional versus DTM SCS. At the end of each programming period, both conventional SCS and DTM SCS significantly reduced back pain NRS from baseline (*P* < .001); the percentage decrease in low back pain NRS from baseline with DTM SCS (68%) was significantly greater compared to conventional (43%; *P* < .0001). An 80% responder rate (response defined as ≥50% pain relief) for low back pain was observed for DTM SCS therapy compared to a 50% back pain responder rate for conventional SCS at the end of each respective programming period. Leg pain NRS was also more significantly reduced from baseline with DTM SCS compared to conventional programming (*P* = .003). More patients (85%) preferred DTM SCS to conventional SCS, and 80% were “very satisfied” or “satisfied” with DTM SCS therapy.^56^

The feasibility study advanced the development of multiplexed programming in 2 ways. First, it allowed clinical testing and optimization of several DTM SCS programs. Based on the programs that patients used, a standardized and consistent DTM SCS approach and algorithm was defined for larger clinical trials. Second, it generated preliminary clinical evidence that DTM SCS offers superior low back pain relief compared to conventional SCS programming. The establishment of a defined starting parameter set and algorithm, in addition to presumed effectiveness, supported the rationale to test DTM SCS against conventional programming in a multicenter RCT.^33^

At the same time as this feasibility study was being conducted, Ruiz-Sauri et al^57^ performed a histological analysis of the human spinal cord. They reported a glial cell to neuron ratio of approximately 12:1 in the posterior gray matter of the T8-T11 vertebral region, increasing to 20:1 in white matter.^57^ While direct clinical evidence of glial cell modulation by DTM SCS is lacking, the abundance of these cells in spinal regions commonly targeted by SCS raises the possibility that these cells may be influenced by neuromodulation. Furthermore, a study of patients with lumbar radiculopathy demonstrated elevated neuroimmune markers (TSPO) in the spinal cord and roots, consistent with glial cell activation.^58^ These markers correlated with pain severity and treatment response, providing clinical evidence that glial activation is a relevant feature of chronic pain. While this study did not assess SCS directly, it supports the plausibility of targeting glial cells in neuromodulation. Given preclinical findings with DTM SCS, hypothesizing that this therapy includes modulation of glial activity is scientifically reasonable and warrants further investigation.

## Defining clinical DTM SCS programming

DTM SCS therapy delivers electrical stimulation to multiple spinal cord regions using distinct electrode configurations and signal types. “Differential target” refers to the spatial targeting of different anatomical areas, while “multiplexed” indicates the concurrent delivery of multiple signals. These core principles—multiplexed signals and spatially distinct anatomical targeting—form the foundation of DTM SCS programming (Box 1).

Box 1. DTM SCS programming for low back and leg pain

1. DTM SCS for low back and leg pain is usually delivered between the mid-T8 to mid-T10 spinal levels. If the lead spans this region after paresthesia mapping, then consider DTM SCS programming.
2. Principle 1: Multiplexed signals
  - Optional starting parameters for DTM SCS programming:
    - A low frequency (base signal) typically 50 Hz as studied in RCTs, but may be ≥20 Hz and <200 Hz and pulse width of 200 µs.
    - A high frequency (prime signal) typically 900 Hz as studied in RCTs, but may be ≥200 Hz and ≤1200 Hz and pulse width of 170 µs.
    - Cycling may be considered as needed for lower energy demand^59^
3. Principle 2: Multiple targets
  - Base and prime programs use different spinal locations for their cathodes.
4. Configure 3 therapy group options (as needed) to allow for quick programming adjustments at adjacent spinal areas. Each therapy group targets a different spinal location to account for anatomical variations in patients.
5. Therapy is titrated based on physician assessment and patient feedback with the goal of providing pain relief and comfortable stimulation.
6. As a best practice, conduct daily patient follow-ups to assess pain relief and adjust for optimal programming during initial therapy set-up.

**Figure 2:**
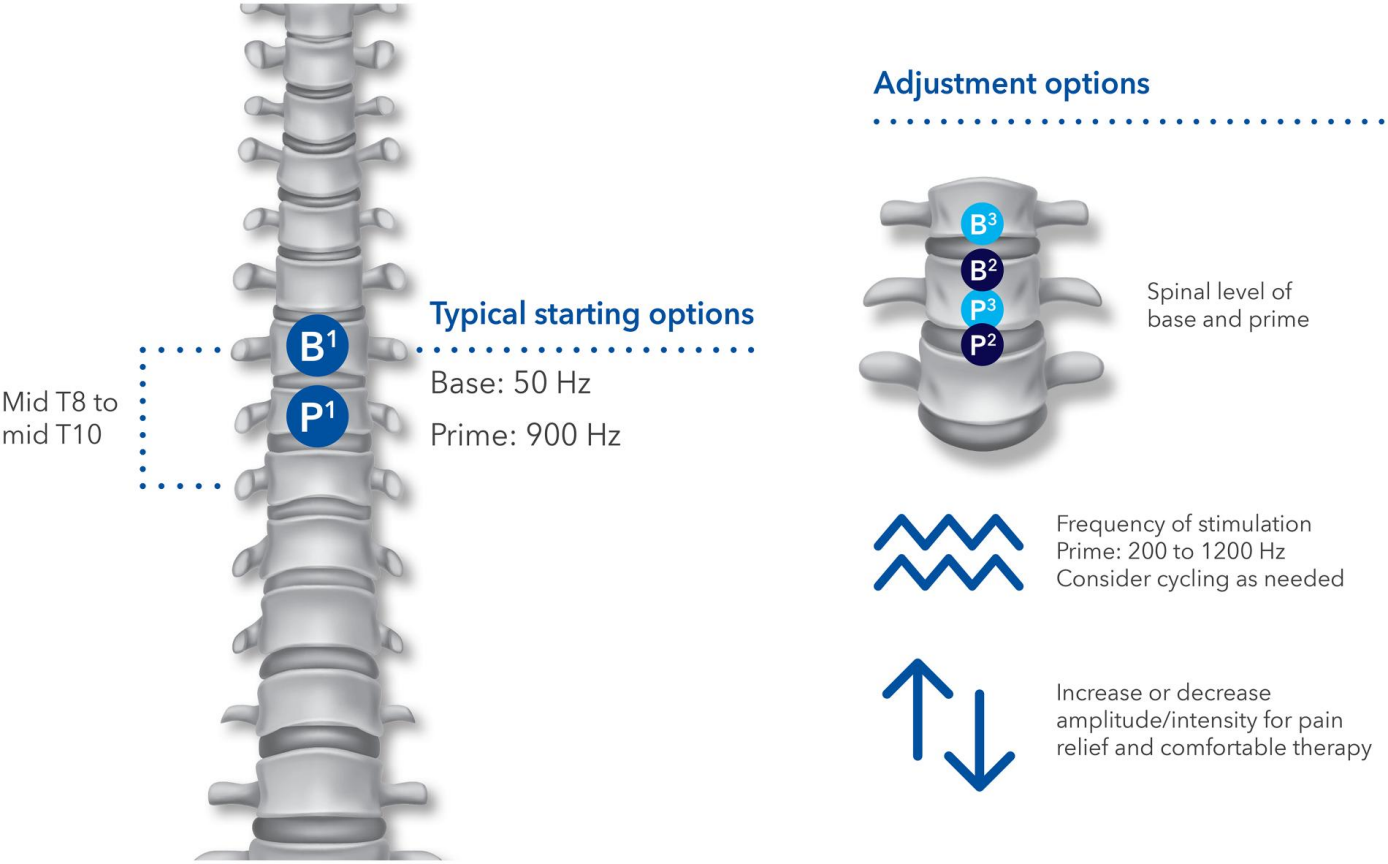
Programming for DTM SCS consists of a base (B) and prime (P) program. For low back and leg pain, therapy is typically delivered in the mid-T8 to mid-T10 range using base and prime signals. The base and prime programs are usually delivered on the same lead and separated by several contacts. The location of the base (B) and prime (P) may be adjusted to target adjacent areas of the spinal cord (B1-P1, B2-P2, or B3-P3) as needed to personalize the therapy for the patient. The frequency of the prime stimulation can be adjusted to tailor therapy to the patient and to lower energy use (typically between 200 to 1200 Hz). Finally, amplitude is adjusted by the patient to ensure comfortable stimulation with desired pain relief.

Following the feasibility study, a more structured DTM SCS programming algorithm was defined. Typically, a low-frequency “base” signal (e.g., 50 Hz, 200 μs) and one or more high-frequency “prime” signals (e.g., 900 Hz, 170 μs) are delivered simultaneously via separate contacts on the same lead, with the prime program generally positioned caudally to the base (Figure 2). This concurrent delivery creates an integrated, synergistic therapy, rather than alternating or independent programs.

Initial lead placement is guided by paresthesia mapping, and programming for low back and leg pain typically occurs between T8 and T10 (Box 1; Figure 2).^33^ The DTM SCS programming strategy is designed to accommodate variability in spinal cord anatomy and the unpredictability of optimal stimulation sites. Up to 3 distinct therapy group options are usually programmed for each patient, with additional options possible depending on system capabilities. Each group consists of a base and one or more prime signals, assigned to electrodes with the prime signal typically caudal to the base. This flexible, algorithmic approach allows clinicians to offer multiple programming options, recognizing that optimal stimulation sites are often determined through clinical testing and patient feedback.

These starting parameters should be further refined to maximize patient comfort and therapeutic efficacy. Fluoroscopic imaging after patient flexion during trialing is recommended to confirm precise lead location before programming.^16^ And documentation of lead location supports future reprogramming if efficacy changes or lead migration are suspected. While current starting parameters are established for low back and leg pain, DTM SCS is adaptable for other pain types and spinal locations, with further guidance anticipated from clinical studies (Table 2).

**Table 2:** RCTs and Prospective DTM SCS studies.

	Citation	Study groups	Indications	Follow-up	Outcome
**RCT 1** **DTM RCT**	Fishman et al (2021)^33^	Conventional SCS vs DTM SCS *N* = 128 randomized	Low back and leg pain PSPS-T1 (21.9%) and PSPS-T2 Indicated for SCS	12 mos	Back pain responder rate: 84%
**RCT 2** **NOVA**	White et al (2024)^31^	Conventional SCS vs DTM SCS *N* = 121 randomized	Low back and leg pain PSPS-T1 (DDD, HD, RPS); Ineligible for spine surgery Indicated for SCS	12 mos	Back pain responder rate: 90%
**RCT 3** **EU DTM RCT**	Kallewaard et al (2024)^32^	CMM vs DTM SCS *N* = 112 randomized	Low back and leg pain PSPS-T1; Ineligible for spine surgery Indicated for SCS	24 mos	Back pain responder rate: 88%
**Prospective** **DTM Endurance**	Peacock et al (2024)^59^	Low energy DTM SCS *N* = 35 implanted	Low back and leg pain Indicated for SCS	12 mos	Decrease in overall pain: 50.4%
**Prospective** **PROCURA**	White et al (2024)^60^	DTM SCS *N* = 46 implanted	Upper limb pain Indicated for SCS	12 mos	Upper limb pain responder rate: 86%

Abbreviations: CMM, conventional medical management; DDD, degenerative disk disease; DTM, differential target multiplexed; HD, herniated disk; mos, months; PSPS, persistent spinal pain syndrome (T1 = type 1, T2 = Type 2); RCT, randomized controlled trial; RPS, radicular pain syndrome.

Typical indications for chronic pain of the trunk and/or limbs: failed back surgery syndrome (FBSS), radicular pain syndrome (RPS) or radiculopathies resulting in pain secondary to FBS or herniated disk, postlaminectomy pain, multiple back operations, unsuccessful disk surgery, degenerative disk disease (DDD)/herniated disk (HD) pain refractory to conservative and surgical interventions.

DTM SCS aims to simplify programming by providing clear, predefined starting parameters within a structured workflow, while maintaining adaptability. Clinicians can tailor amplitude and explore alternative therapy groups as needed, supporting individualized care without compromising the core principle of delivering spatially and temporally multiplexed pulsed signals as a unified therapy.

## DTM SCS RCT

The initial DTM SCS RCT was a prospective, post-market US-based study comparing DTM SCS to conventional SCS in patients with chronic low back and leg pain.^33^ The primary objective was to demonstrate non-inferiority, with a secondary analysis testing for the superiority of DTM SCS therapy compared to conventional SCS. Patients were required to have a back pain score of ≥5.0 cm on the 10.0 cm visual analog scale (VAS), with moderate to severe leg pain. About 66% of the enrolled subjects had predominant low back pain (DTM SCS: 72%; conventional SCS: 61%).

**Figure 3:**
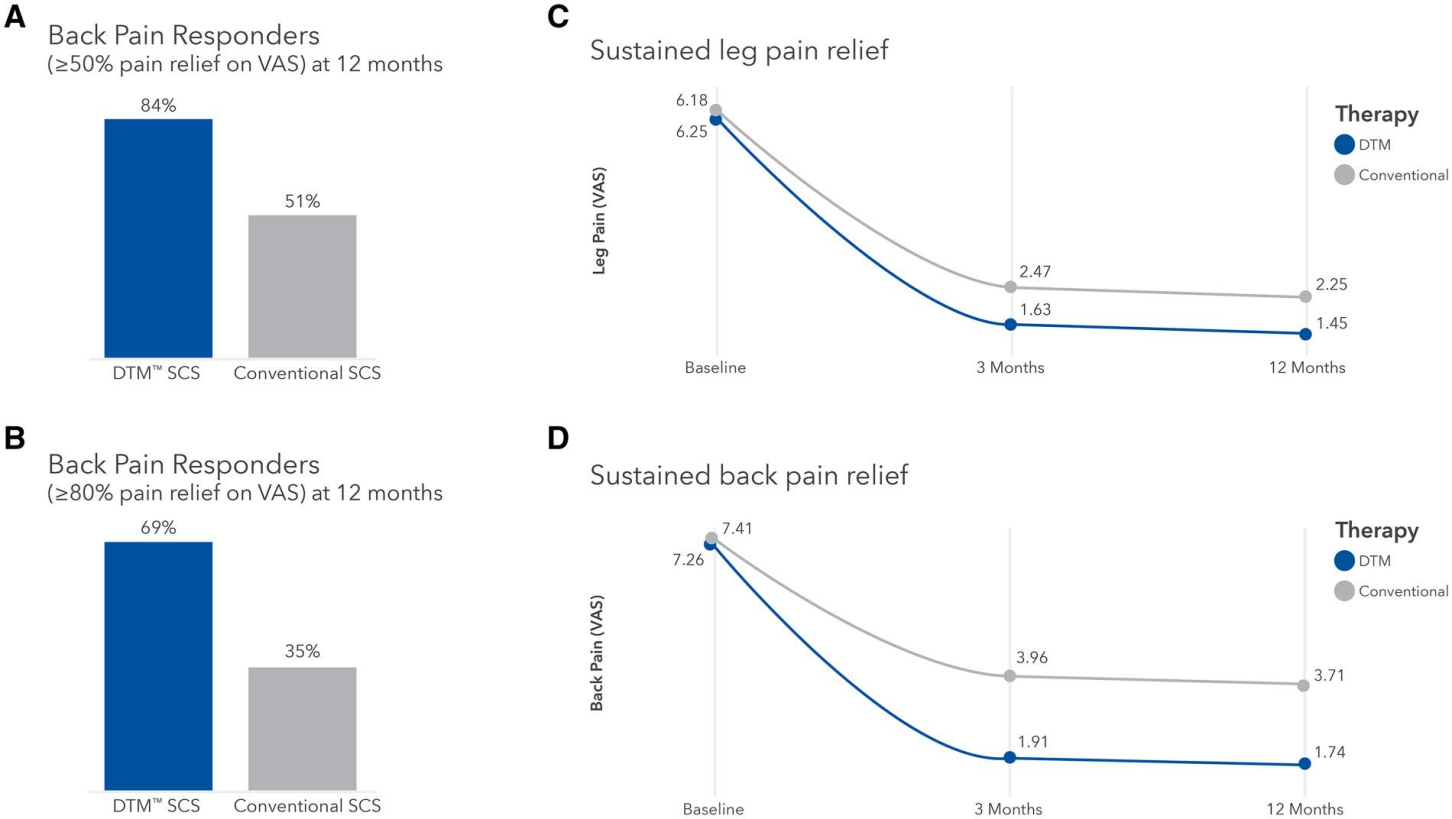
DTM SCS RCT results for low back and leg pain. Patients were randomized to either conventional SCS therapy or DTM SCS therapy for 12 months. (A) The responder rate (≥50% reduction in pain) for low back pain was 84% and 51%, respectively, for DTM SCS and conventional SCS. (B) Profound responders were classified as patients having greater than 80% back pain relief. The profound responder rate was 69% in the DTM SCS group and 35% in the conventional SCS group. (C) Leg pain relief was sustained in both groups through 12 months. (D) Low back pain relief was sustained in both groups through 12 months. DTM SCS decreased back pain intensity more than conventional SCS.

A total of 128 subjects were randomized to either DTM SCS (*n* = 67) or conventional SCS (*n* = 61). After the SCS screening trial, 94 subjects were implanted (47 in each group), and 92 subjects (46 from each group) completed the 3-month primary endpoint. The intent-to-treat analysis at 3 months showed a significantly higher low back pain responder rate (percentage of subjects with ≥50% pain relief) with DTM SCS (80.1%) compared to conventional SCS (51.2%; *P* = .00010). These results were sustained through 12 months (DTM SCS: 83.7%, conventional SCS: 51.1%; Figure 3). Mean VAS reduction for low back pain at 3 months was 5.35 cm (SD 2.63) with DTM SCS and 3.37 cm (SD 2.52) with conventional SCS.

A notable analysis evaluated “profound” responders (≥80% pain relief), a response level that has historically been difficult to capture in large numbers.^2^^,^^61^ At the 3-month follow-up visit, the DTM SCS group demonstrated a profound responder rate of 63% compared to 28% in the conventional SCS group. At the 12-month visit, a profound response was shown in 69% of patients in the DTM SCS group and 35% in the conventional SCS group (Figure 3).

DTM SCS therapy also effectively reduced leg pain. Mean VAS reductions at 3 months were 5.29 cm (SD 2.41) for DTM SCS and 4.76 cm (SD 2.52) for conventional SCS. Leg pain responder rates (≥ 50% leg pain relief) at the 3-month visit were 77.1% in the DTM SCS group and 72.5% in the conventional group, which were sustained through the 12-month visit (DTM SCS: 80.0%; conventional SCS: 75.0%).

Secondary outcomes support holistic patient benefits. The percentage of patients reporting minimal or moderate disability on the Oswestry Disability Index (ODI) questionnaire improved from 27% at baseline to 76% at 12 months in the DTM SCS group, and from 25% to 62% in the conventional SCS group. Patient perception of quality of life, measured with the Patient-Reported Outcomes Measurement Information System (PROMIS) Scale v1.2-Global Health, increased from 39% of patients reporting that their quality of life was “Excellent,” “Very Good,” “Good,” or “Fair” at baseline to 88% of patients at 12 months with DTM SCS. The percentage also increased for conventional SCS, from 36% at baseline to 76% at the 12-month follow-up. The percentage of subjects who were “very satisfied” with DTM SCS therapy was 41.3% at 3 months and 61.9% at 12 months. Satisfaction ratings of “very satisfied” were reported in 39.1% and 45.9% of the conventional SCS patients at the 3- and 12-month visits, respectively. By the 12-month visit, 42.9% of patients in the DTM SCS group and 29.7% in the conventional SCS group reported feeling “a great deal better” as reported on the Patient Global Impression of Change (PGIC).

These findings establish DTM SCS therapy as an effective, long-term solution for chronic low back pain, with superior outcomes to conventional SCS. Conventional SCS programming also provides sustained, long-term benefits for both low back and leg pain. It is a valuable and effective therapy option for patients with chronic low back and leg pain. Clinically, DTM SCS is a logical starting therapy choice for patients with chronic low back and leg pain, with conventional SCS as an additional therapy option if needed.

The study also recognized several limitations. Blinding of the subjects was not feasible due to the nature of the programming, although efforts were made to reduce bias. For example, to try to minimize programmer bias directed toward a single therapy, each SCS group was programmed under the direction of physicians with support of different clinical representatives (i.e., the sponsor’s representatives supporting DTM SCS and device manufacturer’s representatives supporting conventional SCS). The exclusion of patients with prior SCS systems may also be seen as a limitation, in that patients failing conventional SCS programming were not tested with DTM SCS therapy. Finally, since the study was not conducted as a crossover, the within-patient results of conventional versus DTM SCS are not known.

## Other RCTs supporting SCS therapy for low back pain

The initial DTM SCS RCT was not the first study to focus on back pain as a primary outcome in patients with chronic low back and leg pain. In a narrative review of SCS for low back pain, Provenzano et al^62^ identified 5 publications reporting on RCTs including a conventional, tonic study arm. Table 3 is modified from that review, with the addition of the DTM SCS RCT results for comparison.^33^ Interestingly, the authors commented that the outcomes for conventional SCS—particularly related to axial back pain—may have improved over the years due to advances in understanding the stimulation target, electrode placement, stimulation parameters, and new device features. For example, the reemergence of T9-T10 as a target area for low back pain,^63^^,^^64^ as well as technical features such as position-adaptive stimulation^65^ and closed-loop stimulation,^29^ are just examples of modifications that may drive improved low back pain relief for conventional SCS therapy.

**Table 3:** Randomized controlled trials of SCS for low back pain (modified from Provenzano et al, 2021^62^).

	Kumar et al 2007^2^ and 2008^68^	Kapural et al 2015^19^ and 2016^69^	De Andres et al 2017^66^	Deer et al 2018^28^	Mekhail et al 2020^29^^,^^70^	Fishman et al 2021^33^
**Type of study**	Conventional vs CMM	Conventional vs 10 kHz/HF	Conventional vs 10 kHz/HF	Conventional vs BurstDR	Conventional open loop vs closed-loop	Conventional vs DTM SCS
**Study name**	PROCESS	SENZA RCT	—	SUNBURST	EVOKE	DTM SCS RCT
**Number of screening trial subjects (*N*)**	100	198	60	121	134	128 (randomized)
**Successful trials (%), defined per publication**	Conventional : 83%	Overall: 90% 10 kHz: 93% Conventional: 88%	Overall: 92% 10 kHz: 90% Conventional: 94%	Overall: 93% Randomized after conventional screening trial	Overall: 90% Closed-loop: 94% Conventional: 85%	DTM: 98.3% Conventional: 87.9%
**Trial subjects progressing to implant (%)**	89%	86%	92%	83%	86%	81.0%
**Patients diagnosed specifically with PLPS (%)**	100%	77%	100%	42%	59%	59%
**Patients without prior back surgery**	0%	13%	0%	NR	NR	21.9%^71^
**Range of follow-up**	6-24 months	12-24 months	12 months	12-24 weeks	12-36 months	12 months
**Pain pattern**	Radicular > axial	Radicular and/or axial	Radicular	Radicular and/or axial	Radicular and/or axial	Radicular and/or axial
**Pertinent inclusion and exclusion criteria**	Inclusion: PLPS; predominant leg pain. Exclusion: predominant back pain.	Inclusion: trunk/limb pain refractory to CMM for >3 months.	Inclusion: PLPS; pain refractory to CMM for >6 months. Exclusion: mechanical low back pain; coexisting chronic pain or neurological disease.	Inclusion: trunk and/or limb pain >60 mm on VAS during 7-day pain diary.	Inclusion: trunk/limb pain refractory to CMM; >60 mm on VAS	Inclusion: trunk and/or limb pain; back pain ≥ 5.0 cm on VAS with moderate to severe chronic leg pain
**Pain scale used**	VAS	VAS	NRS	VAS	VAS	VAS
**Back pain (% reduction at last follow-up, if reported)**	13%	10-kHz HF: 67% conventional: 44%	NR	Burst 5.7 mm less than conventional on 100 mm VAS	12-months Closed-loop: 69% Conventional: 54% 36-months: NR	DTM: 75.2% Conventional: 49.0%
**Leg Pain (% reduction at last follow-up, if reported)**	46%	10-kHz HF: 70% Conventional: 49%	NR	Burst 4.7 mm less than conventional on 100 mm VAS	12-months Closed-loop: 73% Conventional: 62% 36-months: NR	DTM: 75.7% Conventional: 67.9%
**Overall Pain (% reduction at last follow-up, if reported)**	NR	NR	10-kHz HF: 24% Conventional: 19%	Burst 5.1 mm less than conventional on 100 mm VAS	36-months Closed-loop: 70% Conventional: 54%	NR
**Back pain responder rate (≥50% pain reduction**	NR	3 months: 10-kHz HF: 84% Conventional: 44% 6 months: 10-kHz HF: 76% Conventional: 52% 12 months: 10-kHz HF: 79% Conventional: 51% 24 months: 10-kHz HF: 77% Conventional: 49%	NR	NR	3 months: Closed-loop: 81% Conventional: 57% 12 months: Closed-loop: 80% Conventional: 58% 36-months (overall back/leg responder) Closed-loop: 78% Conventional: 49%	3 months: DTM: 80.1% Conventional: 51.2% 6 months: DTM: 73.9% Conventional: 50.0% 12 months: DTM: 83.7% Conventional: 51.1%
**Patient satisfaction (satisfied and very satisfied, unless noted)**	93%	10-kHz HF: 83% Conventional: 79%	NR	89%	NR	DTM (very satisfied): 61.9% Conventional (very satisfied): 45.9%
**SCS complications**	LM (14%) LOP (12%) PIS (12%) INF (10%)	PIS (12%) INF (7%) LM (5%)	LM (13%)	ULP (0.6%) PIS (0.6%)	LM (7%) PGPP (4%) MS/C (2%) USL (2%) INF (1%)	Abd pain (0.8%) Implant site irritation (0.8%) PGPP (0.8%) Pain (0.8%) Implant site INF (0.8%) Wound INF (0.8%) PIS (0.8%) Pneumocephalus (0.8%) Proc Comp (0.8%) LD (1.6%) Pruritus (0.8%)
**Comments**	At 24 months, PLPS patients reported sustained pain relief, improvements with functional capacity and health-related quality of life, and satisfaction with treatment.	Results indicate that 10-kHz high-frequency therapy is superior to conventional SCS.	Results indicate that PLPS can be treated with similar effectiveness by conventional or 10-kHz therapy.	Purpose of study was to determine safety and efficacy of a device capable of delivering tonic or burst stimulation.	Evidence indicates superiority of closed-loop (ECAP controlled) system.	Results indicate that DTM SCS therapy is superior to conventional SCS.

All percentages rounded to whole numbers unless <1%.

Abbreviations: 10-kHz HF, 10 kHz high-frequency therapy; Abd, abdominal; CMM, conventional medical management; ECAP, evoked compound action potential; INF, infection; LM, lead migration; LOP, loss of paresthesia; N MS/C, muscle spasm/cramps; RS, numeric rating scale; PGPP, pulse generator pocket pain; PIS, pain at incision site; PLPS, postlaminectomy pain syndrome (ie FBSS, failed back surgery syndrome); Proc Comp, procedural complication; ULP, unsuccessful lead placement; SCS, spinal cord stimulation; USL, unintended stimulation location; VAS, visual analog scale.

In those articles comparing a new SCS modality to conventional SCS, most were able to demonstrate the superiority of the new modality over the conventional therapy. Provenzano et al^62^ pointed out that many of these studies were “industry sponsored with heterogeneous patient populations often with radicular symptoms and history of prior back surgery,”—factors that may be essential limitations.^62^ In a “risk of bias” assessment that included several of these studies, the publication by De Andres et al^66^ was determined to have the lowest risk of bias, due to the use of a blinded assessor and low bias in other categories.^67^ Bias may also have been lessened as a physician-sponsored study, free from industry support.

## DTM SCS: expanding patient access

### DTM SCS low energy derivatives

Leveraging the foundational DTM SCS research, a reduced-energy derivative of DTM SCS was studied to expand DTM SCS access to patients who needed lower energy demands. First, preclinical rodent studies showed that biological and behavioral responses to reduced-energy programming were similar to the original DTMP programming.^72^^,^^73^ Next, a feasibility study also found that switching from traditional DTM SCS to the low-energy derivative provided equivalent pain relief with significantly less energy consumption.^74^ Finally, this approach, termed DTM Endurance, was further investigated in the DTM Endurance study, a prospective single-arm trial of 35 implanted patients.^59^

The DTM Endurance study reduced the prime frequency to 200-250 Hz, the base frequency to 10-60 Hz, and enabled therapy cycling.^59^ The initial cycling ratio was 1:2 (15 minutes on:30 minutes off), later adjusted as needed to 1:1 (15 minutes on:15 minutes off to start). By the 12-month follow-up, most patients (63%) remained on 1:2 cycling. The primary outcome—overall pain reduction measured by VAS—showed a mean decrease of 3.9 (SD 2.5) at 3 months, from 7.8 (SD 1.1) at baseline to 3.8 (SD 2.4). At 12 months, overall pain decreased by 4.4 cm (SD 2.8), with sustained reductions in back and leg pain of 4.2 cm (SD 2.9) and 4.7 cm (SD 2.9), respectively.

Device longevity and recharge modeling, based on the programming parameters used by subjects in the study and accounting for impedance, projected mean primary cell device lifespans of 5.8 (0.7) to 6.8 (0.6) years.^59^ For rechargeable systems, recharge modeling using a 900 Ohm saline bath estimated 60 minutes of recharge, a mean (SE) of every 11.5 (0.6) days, or a mean (SE) of 5.9 (0.5) minutes of daily recharge. While these longevity calculations were based on actual programming parameters used by subjects in the study, it should be appreciated that they are projections, and device longevity for patients will vary.

The DTM Endurance study provided practical data and longevity expectations for this reduced-energy therapy, helping meet the needs of patients with recharge-free neurostimulators or those seeking longer recharge intervals. Unlike some primary cell devices with 10-year claims—achievable only with rarely used settings (0.6 mA, 500 Ohms, and a duty cycle of 30 s on/360s off)—predicted DTM Endurance longevity is based on modeling of real-world patient programming and individual impedance, which are crucial for accurate lifespan prediction.^75^

### DTM SCS for chronic low back pain in subjects ineligible for surgical spine treatment

The first DTM SCS RCT enrolled patients with low back and leg pain from a variety of etiologies, including PSPS-T2 and PSPS-T1. A sub-analysis of PSPS-T1 subjects showed meaningful and sustained low back pain relief out to 12 months.^71^ To further strengthen the evidence, 2 additional RCTs have investigated DTM SCS in patients with chronic low back pain who are not eligible for spine surgery (Table 2).

White et al^31^ published the NOVA RCT (NCT04571242), sponsored by SGX NOVA LLC (acquired by Medtronic). The study investigated the use of DTM SCS therapy for patients with chronic, intractable low back pain without prior history of spine surgery and who were deemed ineligible for spine surgery treatment. Patients were indicated for SCS per Medtronic labeling in the United States with indications of radicular pain syndrome, degenerative disc disease, or herniated disc-related back pain. This open-label, multicenter study randomized subjects to DTM SCS (*n* = 51) or conventional SCS (*n* = 54). At the 3-month primary endpoint, DTM SCS achieved a back pain responder rate of 93.5% versus 36.4% conventional SCS (*P* < 0.0001), with superior responder rates sustained at all time points. Back pain VAS scores decreased in the DTM SCS group from a baseline of 7.90 cm to 1.44 cm at 3 months and remained low through 12 months (1.46 cm); in the conventional SCS group, the baseline VAS score was 8.00 cm, decreasing to 4.90 cm at 3 months and 5.70 cm by 12 months.

An optional crossover was available to all participants at 6 months. No DTM SCS subjects chose to crossover, while 14 of 30 subjects (46.7%) with conventional SCS chose to crossover to DTM SCS. One subject withdrew from the study; of the remaining 13 subjects, 12 were responders at 9 and 12 months (≥50% low back pain relief). In these crossover subjects, VAS back pain scores had reduced by 2.33 (2.75) cm using conventional SCS, but reduced by 5.95 (2.24) cm with DTM SCS at 12 months. These results highlight the ability of DTM SCS to provide superior and lasting pain relief, even for patients not responding to conventional SCS.

At 12 months, DTM SCS maintained a back pain responder rate of 90.5%, with 92.5% of patients “very satisfied” or “satisfied” with the therapy. Conventional SCS resulted in a back responder rate of 25.0% at 12 months, yet most of the remaining subjects (*n* = 14) were still “very satisfied” or “satisfied” with the therapy (85.7%). DTM SCS also improved disability with ODI scores (SD) decreasing from 50.3 at baseline to 23.5 (16.1) at the 12-month visit. The adverse events (AEs) and serious AEs reported in the study were consistent with reports from other SCS studies, and there were no device-related serious AEs. Both treatment groups had a similar AE profile and demonstrated an acceptable risk profile. This RCT demonstrated the superior back pain relief provided by DTM SCS over conventional SCS in PSPS-T1 patients with radicular pain syndrome, degenerative disc disease, or herniated disc-related back pain deemed not eligible for spinal surgery treatment.

A second RCT (ISRCTN10663814) sponsored by SGX International LLC (acquired by Medtronic) was conducted on a similar patient population in Europe.^32^ This study compared DTM SCS (*n* = 55) to conventional medical management (CMM; *n* = 57) in SCS-indicated PSPS-T1 patients ineligible for spine surgery.^32^ The study was conducted in accordance with European SCS labeling and regulations; therefore, it represents a population of PSPS Type 1 patients slightly broader than the population in the NOVA study. DTM SCS had a 6-month responder rate (≥50% low back pain relief) of 86% versus 4% for CMM. Back pain responder rate was 80.0% at 12 months and 88.4% at 24 months (modified intention-to-treat analysis). Subjects were provided the option to crossover to the alternate therapy at the 6-month visit, and 50 CMM subjects chose to cross over to DTM SCS. No patients chose to crossover from the DTM SCS to the CMM arm. A per-protocol analysis reported a back pain responder rate of 90.2% and 97.4% for the 6- and 12-month visits, respectively, in the crossover population. Disability (ODI) and quality of life (EQ-5D-5L) improved substantially at 12 months, with high satisfaction responses including 70.7% reporting “very satisfied” with DTM SCS and an additional 17.1% “satisfied” with the therapy. A total of 84.2% of patients reported feeling “very much improved/much improved” with DTM SCS at the 12-month visit. Adverse event rates were as expected for both treatments.

Together, these 2 RCTs support the use of DTM SCS therapy in specific subsets of patients who have not had prior spine surgery and who are not candidates for surgical treatment, including patients with back pain related to degenerative disc disease.

Historically, data for SCS for patients with PSPS-T1 were limited, but confidence is growing with 5 RCTs focused on similar patient populations (Table 4). Four RCTs have compared different modalities of SCS to CMM, with responder rates for pain/back pain ranging from about 73% to 90% in the SCS arms. The NOVA study is the only RCT to compare conventional SCS to another SCS modality (DTM SCS). DTM SCS resulted in significantly greater and superior reduction of low back pain in significantly more patients than conventional SCS. Among patients who crossed over from conventional SCS to DTM SCS, most responded with substantial pain improvement. Table 4 summarizes information across these studies (DTM-related studies are shaded); note that the specific indications vary across manufacturers.

**Table 4:** Randomized controlled trials of SCS for low back pain in patients without prior spine surgery (PSPS Type 1).

	White et al 2024^31^	Kallewaard et al 2024^32^	Kapural et al 2022^27^ and Patel et al 2023^34^	Deer et al 2023^30^ and Yue et al (2024)^76^	North et al 2025^77^
**Type of study**	Conventional SCS vs DTM SCS	CMM vs DTM SCS	CMM vs 10 kHz/HF	CMM vs BurstDR	CMM vs multiple SCS options
**Study name**	NOVA	EU DTM SCS RCT	SENZA NSRBP	DISTINCT	SOLIS
**Total randomized**	121 (conventional SCS: 54/DTM SCS: 51; not trialed: 16)	112 (CMM: 57/DTM SCS: 55)	159 (CMM: 76/10 kHz SCS: 83)	269 (CMM: 107/BurstDR: 162)	147 (CMM: 68/multiple SCS options: 79)
**Number of screening trial subjects (*N*)**	Conventional: 54 DTM SCS: 51	DTM SCS: 51	10 kHz SCS: 80	BurstDR: 142	SCS: 77
**Successful trials (%)**	Conventional SCS: 80% DTM SCS: 96%	DTM SCS: 94%	10 kHz SCS: 93%	BurstDR SCS: 88%	SCS: 92%
**Trial subjects progressing to implant (%)**	Conventional: 60% DTM SCS: 88%	98%	93%	92%	89%
**Patients without prior back surgery**	100%	100%	100%	100%	100%
**Follow-up**	12 months	24 months	24 months	12 months	12 months
**Pertinent inclusion and exclusion criteria**	Inclusion: labeled indication (back pain with or without leg pain); chronic, refractory axial low back pain with a neuropathic component and not eligible for spine surgery; average low back pain intensity ≥6 cm on the 10 cm VAS Labeled indications: DDD or HD refractory to conservative and surgical interventions or patients with radicular pain syndrome.	Inclusion: refractory axial low back pain with or without lower limb pain with a neuropathic component as assessed by the investigator; 6 months refractory to conventional therapy; not eligible for spine surgery; average back pain intensity of ≥6 out of 10 cm on the VAS	Inclusion: diagnosis of chronic, axial, low-back pain with a neuropathic component and no previous spine surgery; a consultation with a spine surgeon deemed the patient inappropriate for spine surgery.	Inclusion: no spine surgery for back or leg pain; chronic (≥6 months) refractory axial low back pain with a neuropathic component and not a candidate for spine surgery; low back pain ≥6 on NRS Exclusion: 1. Pathology seen on imaging tests obtained within the past 12 months that is clearly identified and is likely the cause of the CLBP, that can be addressed with surgery; primary symptom of leg pain, or leg pain is greater than back pain	Inclusion: chronic predominant low back pain, with or without leg pain, for at least 6 months; average pain of 6 or greater; diagnosis of axial lumbar degenerative disc disease with/without lumbar spinal stenosis
**Pain responder rate (≥ 50% pain reduction**	Back pain responder rate: 3 months: Conventional: 36% DTM SCS: 94% 6 months: Conventional: 28% DTM SCS: 88% 9 months: DTM SCS: 88% Conventional: 25% 12 months: DTM SCS: 90% Conventional: 25%	Back pain responder rate: 3 months: CMM: 5% DTM SCS: 85% 6 months: CMM: 4% DTM SCS: 85% 12 months: DTM SCS: 80% 24 months: DTM SCS: 88%	Pain responder rate: 3 months: CMM: 1% 10-kHz HF: 81% 6 months: CMM: 3% 10-kHz HF: 80% 12 months: 10-kHz HF: 78% 10 kHz HF/crossover: 78% 24 months: 10-kHz HF: 88%	Back pain responder rate: 6 months (ITT)^30^ CMM: 6% BurstDR: 73% 12 months: BurstDR: 78.6% CMM crossover to SCS, 6 months: BurstDR: 71.4%	Responder rate, overall pain 3-months (mITT): CMM: 8.1% SCS: 89.5% 12-months: SCS: 86%
**Patient global impression of change (PGIC)**	88.7% PGIC, very much/much improved (12 months) with DTM SCS	93% PGIC, very much/much/minimally improved (24 months) with SCS	76% PGIC, better or a great deal better (24 months) with SCS	88% PGIC, moderately better, better, or a great deal better (12 months) with SCS	82.5% (SCS) and 78.5% (CMM crossover to SCS) PGIC, very much/much improved (12 months)

Specific indications vary across manufacturers and geographies.

Abbreviations: 10-kHz HF, 10 kHz high-frequency therapy; CLBP, chronic low back pain; CMM, conventional medical management; DDD, degenerative disc disease; HD, herniated disc; NRS, numeric rating scale; SCS, spinal cord stimulation; VAS, visual analog scale.

### DTM SCS for upper limb pain

Expanding SCS therapy to indications beyond back and leg pain increases patient access. White et al^60^ published a prospective, open-label, single-arm, multicenter study on DTM SCS for patients with chronic upper limb pain (NCT04466111). The primary pain-related etiology reported in the study was cervical radiculopathy (86.5%). Of the 58 subjects enrolled, 52 completed an SCS trial; 94% were responders, and 46 received a permanent implant. The 3-month primary endpoint responder rate was 91.5% (≥50% upper limb pain relief), which was maintained through the course of the 12-month follow-up with a responder rate of 86.0%. Upper limb pain scores (VAS) decreased by over 79% at all follow-up points. At the 12-month visit, almost 95% of subjects were “very satisfied” with DTM SCS therapy, and nearly 95% reported “very much improved/much improved” on the PGIC.

No unanticipated AEs related to procedure or device occurred; most of the 16 reported AEs were mild (*n* = 12). No uncomfortable sensations due to neck motion were reported as an AE. Spinal cord bruising was the single serious AE reported. The publication reported that the device was explanted and the subject was still undergoing medical treatment. The Neuromodulation Appropriateness Consensus Committee (NACC) recommended using imaging for preprocedural planning and careful selection of the percutaneous epidural entry site for cervical SCS lead placement. The NACC also recommended that the level of percutaneous epidural entry site for cervical SCS lead placement be chosen based on pain location, anatomical variations, and physician experience.

### Limitations

DTM SCS is supported by hypothesis-driven preclinical research, and like all SCS modalities, its mechanisms remain difficult to confirm clinically. However, differences in patient outcomes—particularly apparent in the NOVA RCT subject crossover from conventional SCS to DTM—suggest a potential difference in modulatory effects between conventional and DTM SCS. Preclinical data point to glial cell modulation and altered neuro-glial interactions as a plausible hypothetical mechanism underlying the observed pain relief. Notably, most preclinical studies on DTM SCS to date have been conducted by a single research lab, underscoring the need for independent replication to validate findings and enhance generalizability. Broader scientific engagement will be essential to confirm these mechanisms and further elucidate the neurobiological effects of DTM SCS.

Numerous waveforms have demonstrated efficacy in improving pain relief, particularly for low back pain (Figure 1; Tables 3 and 4). Most large RCTs have compared novel waveforms to conventional SCS or CMM, providing valuable comparative data relative to the historical standard of care. However, there is a general lack of RCT-level comparative studies between more recent waveforms. For example, DTM SCS has not been prospectively compared to 10 kHz or BurstDR waveforms in a randomized manner.

## Conclusions

The DTM SCS journey illustrates a successful rational development pathway from a preclinical hypothesis to human feasibility concept testing to evaluation in 3 RCTs. DTM SCS was identified early in preclinical studies as uniquely effective in reversing pain behaviors and modulating both neuronal and glial cell activity, supporting a mechanistic hypothesis of glial-neuronal interaction. These findings demonstrate that DTM SCS engages the nervous system in a manner distinct from low-rate or high-rate SCS alone. The application in humans was vetted and calibrated through feasibility testing before being tested in an initial RCT. The RCT helped to define a systematic approach, with programming guidance for parameters and electrode/contact targets. In addition to showing strong efficacy for low back pain relief, the use of an algorithmic or workflow-based approach simplifies the application and programming of DTM SCS.

The first RCT also established the superiority of DTM SCS over conventional SCS therapy for the treatment of chronic low back pain and invited the testing of DTM SCS for other pain conditions, including low back pain in PSPS-T1 patients ineligible for spinal surgery treatment and upper limb pain. DTM SCS has now been evaluated in 3 RCTs for patients with chronic low back and leg pain, with and without prior spine surgery, firmly establishing its place as a treatment for low back pain. DTM SCS continues to be tested for other pain areas and indications, including radicular pain of the upper limb and diabetic peripheral neuropathy. Lower energy DTM settings have also shown sustained pain relief for chronic back and leg pain patients out to 12 months. All clinical evidence presented here on DTM SCS has been established with open-loop delivery. Testing of DTM SCS with features that help to personalize and optimize therapy, such as closed-loop technology, is also ongoing. The ability to continue to evolve therapy programming based on animal models is forward-thinking and will allow for future refinement to specific disease states.

## Disclosures

This supplement was sponsored by Medtronic, which provided funding for its publication. No authors received compensation for their contributions to the writing, review, or critical input into the content of this article. J.G. and L.J. are Medtronic employees and participated in the manuscript writing and editing. All authors critically reviewed the manuscript and approved the final version.
